# The combination of breast necrosis and chylothorax following the OPCAB

**DOI:** 10.15171/jcvtr.2016.18

**Published:** 2016-06-30

**Authors:** Feridoun Sabzi, Alireza Yaghoubi

**Affiliations:** ^1^Department of Cardiovascular Surgery, Imam Ali Heart Center, Kermanshah University of Medical Sciences, Kermanshah, Iran; ^2^Rajaie Cardiovascular Medical and Research Center, Iran University of Medical Sciences, Tehran, Iran

**Keywords:** Coronary Artery Bypass Grafting, Complication, Breast Necrosis

## Abstract

Due to long term patency, the internal mammary artery is considered as a conduit of choice for revascularization of the left anterior descending coronary artery. The internal mammary artery and its accessory branches in addition to perfusing the chest wall structures also contributes to supplying, part of the female breast arteries. In addition, due to the accompaniment of thoracic duct branches with the left internal mammary artery, harvesting may be associated with injury to these branches and contribute to chylothorax. We report a rare case of chylothorax and the breast necrosis following the coronary artery bypass grafting. The chylothorax was started in the second postoperative day and ceased gradually in the 12th day of operation. The breast necrosis appeared in the 3th weeks of operation with pain, and tenderness and black skin color change. The patient underwent total mastectomy in the 4th weeks of operation.

## Introduction

The blood supply of the breast arises primarily from both left and right thoracic internal mammary arteries (LIMA,RIMA) intercostal artery and the subclaveian artery. These arteries runs below the pectoralis muscles and overlying breast and the chest wall muscles. The internal thoracic arteries not only provide nutrients and oxygen, to the breast tissue but also accompany its specific lymph vessels that provide lymph drainage of thoracic organs between them is lung tissue. The damage to branches of thoracic lymph ducts during harvesting may be associated with chylothorax.^1^ In women undergoing coronary artery bypass grafting (CABG) with LIMA use, breast necrosis is more likely to occur in women who have huge breast tissue, atherosclerotic risk factors such as chronic hypertension, diabetes, and hyperlipidemia. In opposed to main LIMA artery that is not involved in the atherosclerotic process, LIMA branches may be involved by atherosclerosis and could be contributing to breast ischemic changes. Perforating branches of LIMA that deeply located in the pectoralis muscles may be also involved in atherosclerosis process could be considered as a risk factor in the necrosis of huge breast, breast of patient with diabetes and microvascular disease or history of smoking or opium using.^2^ Necrosis of the breast is an exceedingly rare phenomenon, accounting for less than 0.001% of all post CABG complications, however the incidence of chylothorax is much higher. Breast necrosis is often limited to a small area in the central zone, but in our patient, due to severe damage to harvesting site, this zone was wider and was associated with chylothorax.^3,4^

## Case Report

**Figure 1:**
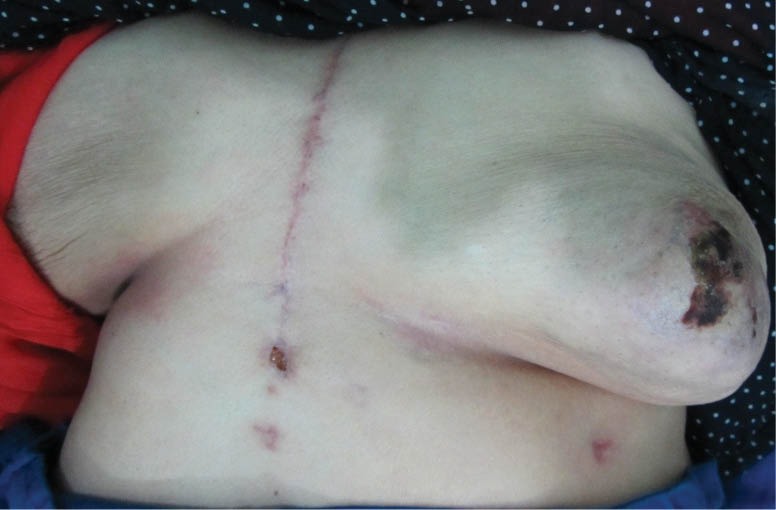
Left breast necrosis following coronary artery bypass grafting

**Figure 2:**
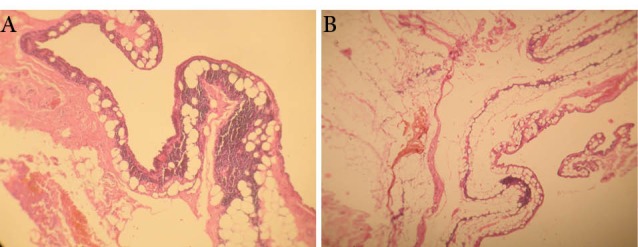
A) Stages of fat necrosis as seen on photomicrographs. Early stage of fat necrosis shows individual adipocytes (arrows) surrounded by areas of hemorrhage. (Papanicolaou, ×40). B) Stages Intermediate fat necrosis as seen on image, that shows conglomeration of RBCs”, which is characteristic of fat necrosis. (Papanicolaou, ×40).

A 68-year-old female admitted to our center with severe chest pain that exaggerated with exertion. Left main coronary artery disease was confirmed by angiography. During the physical exam no evidence of lump or others skin legion were found. There was also no history of trauma, surgery or irradiation to the breast. The patient had a history of a diabetes, hypertension and smoking. A chest x-ray was within normal limits. The CABG was performed by OPCAB method and use of conduit grafts such as saphenous graft and LIMA. LIMA harvesting and preparation was performed by training resident, using a similar surgical technique in our center. Following median sternotomy, the left half of sternum was elevated using IMA Retractor. The pleura was opened longitudinally according to subject’s discretion. The IMA routinely harvested as a pedicled graft, a large pedicle containing the IMA, accompanying with double veins, endothoracic fascia, fat, and lymphatics tract and lymphatics node and some times, thin layer of intercostals muscle that was mobilized using low voltage bipolar electrocautery. Using the tip of the electrocauter as a dissector, the branches of the IMA were exposed, clipped proximally and distally, and transected using thin scissors. Revascularization of the left anterior descending artery (LAD) with the LIMA was typically performed first, followed by revascularization of LCX and the right coronary artery distribution. The postoperative period was complicated by chylothorax Peripheral smear of lymph also shows free fat in microscopic exam. The fat content of lipid (triglyceride: 320 mg/dL) was high in the blood serum level. Lymphocytes were the main cellular component of lymph by in the range of 5,000 per mm^2^ The Red blood cells content was low and theirs range was between 20-50 cells per mm^2^. The initial treatment of chylothorax was conservative approach, to minimize chyle formation, to prevent the immune deficiency, as well as to replace a high-fat diet with the highest content protein diet. The drainage gradually decreased with conservative therapy, and completely ceased in the 10th day of operation and the patient discharged on the 11th post-operative day. The patient complained of the left anterior thoracic pain that attributed to the pain of sternotomy and treated with nonsteroidal anti-inflammatory drug. No evidence of breast skin color change was detected. The patient came back to the clinic with breast pain and a black necrotic crater over the left breast in third weeks of surgery. There was not a history of discharge from the sternotomy incision or evidence of dehiscence. On local examination, there was a healed sternotomy scar; a tender indurate mass was appreciated on the central and left upper quadrant of the left breast with overlying skin necrosis (Figure 1). No palpable axillaries nodes were noted. Needle aspiration did not reveal any collection; cytology was reported as inflammatory cells with no evidence of malignancy. The mass and local symptoms persisted with no response to antibiotic therapy for one week. The lesion was examined by a general surgeon and he planned a total mastectomy that was performed two days later at another hospital. The cut surface of the breast appeared as a necrotic and hemorrhagic mass. Microscopic examination of the breast demonstrated early stage of fat necrosis that shows individual fat cells surrounded by areas of hemorrhage. And in later stages of fat necrosis was seen as a conglomeration of RBCs, which is characteristic of fat necrosis (Figure 2A and 2B). She discharged in the third day of surgery. In the sixth month of follow-up, the mastectomy incision site has been healed and evidence of mass or subcutaneous hematoma or seruma were not detected.

## Discussion

Due to abundant collateral arteries arises from six important branches of subclaveian and axillary artery, the complications of LIMA harvesting are rare after CABG, but huge breast size in any rare case may be perfused selectively from lima artery and its inappropriate harvesting with large pedicle may predispose the breast to necrosis. In other hand risk factors such as hypertension, diabetes and smoking and renal failure may aggravate the atherosclerotic change in the deeply located perfusing arteries.^5^ In addition to breast necrosis another complication of LIMA harvesting includes chylothorax and others type of plural effusions. The post CABG chylothorax has many etiologies, such as LIMA, RIMA harvesting, thymus handing, ascending aortic clamping, snaring of the SVC or IVC, pulmonary hypertension.^6^ The incidence of post-CABG breast necrosis is very low in the medical literature that none of them occurred in combination with chylothorax. If we consider the huge number of CABG that performed annually in word, the reported cases of chylothorax is an exceedingly rare complication. The rarity of this complication in OPCAB could be explained by lower chance of injury to the thoracic duct during surgery. In opposed to off-pump surgery some additional procedures may be performed in on-pump CABG such as ,snaring of SVC, handling of ascending aorta, aortic cross clamping that may further increase the risk of chylothorax.^7^ Other possible etiologies have been proposed, such as increased superior vena cava pressure due to use of tapes or to venous thrombosis during cardiopulmonary bypass. In addition, injury to a large branch of thoracic duct may be occurred during dissection of thymus or LIMA harvesting close to subclaveian artery.^8-15^

## Conclusion

Our patient was a smoker, opium addicted, hypertensive and diabetic case that also had a large breast, the combination of these risk factor associated with unknown anomaly of breast supplying arteries may provided an appropriate basis that aggravate with invasive LIMA harvesting in occurrence of breast necrosis. We believe that in patient with aforementioned risk factors the best strategy to prevent or reduce the risk of this complication is LIMA harvest with skeletonized.

## Ethical approval

The study was approved by the Local Ethics Committee.

## Competing interests

Authors declare no conflict of interests in this study.
